# Spatial Estimation of Sub-Hour Global Horizontal Irradiance Based on Official Observations and Remote Sensors

**DOI:** 10.3390/s140406758

**Published:** 2014-04-11

**Authors:** Federico-Vladimir Gutierrez-Corea, Miguel-Angel Manso-Callejo, María-Pilar Moreno-Regidor, Jesús Velasco-Gómez

**Affiliations:** ETSI Topography, Geodetics and Cartography, Universidad Politécnica de Madrid, Campus Sur UPM, Autovía de Valencia Km 7.5, E-28031 Madrid, Spain; E-Mails: m.manso@upm.es (M.-A.M.-C.); mariapilar.moreno@upm.es (M.-P.M.-R.); jesus.velasco@upm.es (J.V.-G.)

**Keywords:** spatial interpolation, 15-minute Global Solar Radiation, volunteer stations

## Abstract

This study was motivated by the need to improve densification of Global Horizontal Irradiance (GHI) observations, increasing the number of surface weather stations that observe it, using sensors with a sub-hour periodicity and examining the methods of spatial GHI estimation (by interpolation) with that periodicity in other locations. The aim of the present research project is to analyze the goodness of 15-minute GHI spatial estimations for five methods in the territory of Spain (three geo-statistical interpolation methods, one deterministic method and the HelioSat2 method, which is based on satellite images). The research concludes that, when the work area has adequate station density, the best method for estimating GHI every 15 min is Regression Kriging interpolation using GHI estimated from satellite images as one of the input variables. On the contrary, when station density is low, the best method is estimating GHI directly from satellite images. A comparison between the GHI observed by volunteer stations and the estimation model applied concludes that 67% of the volunteer stations analyzed present values within the margin of error (average of ±2 standard deviations).

## 1. Introduction

Global Horizontal Irradiance (GHI) sensors are especially relevant as a source of information for calculating numerous ecological and industrial processes (*i.e*., photosynthesis, evaporation-transpiration, solar energy production, monitoring SmartCities [1]) and also has other applications including model verification, data inference and data assimilation within models in contexts such as meteorology, climate or hydrology, among other fields of study. In some cases, the complexity of these applications requires an extensive record of observations or increasingly more frequent periodicity and reduced latency between reports [2–4]. Solar Radiation (SR) on the Earth's surface may be measured through direct on-site observation or estimated using an indirect method. Direct observations are made by SR sensors installed in weather stations; however, their scarce spatial densification presents a disadvantage. According to [5], in the US, the proportion of official stations with temperature and SR sensors is 1:100 and, in the rest of the world, it is 1:500. Indirect sources for estimating SR include deriving this information from satellites, determining it based on other meteorological variables and applying spatial interpolation algorithms of point values, among others [5,6].

Despite the various sources used to obtain surface SR values, the best way to measure it is through surface weather stations [7] and some of complex SR applications/processes specify as much. This implies that spatial densification of surface stations is the optimal solution for selecting various sources of weather data. However, according to [8], low densification is due to the fact that the investment required in order to install and maintain these stations has a deterrent effect when it comes to condensing the networks.

The current study uses sub-hour GHI observations for the year 2011 on horizontal surfaces in mainland Spain and the Balearic Islands. Said observations were made by three station networks: two official (AEMet and CASTILLA y LEÓN (CYL)) and one volunteer (Meteoclimatic) with a total of 211 stations. Because of their official status, the first two networks are used in order to better identify the best method for estimating 15-minute GHI in the study area, taking into account the various forms of grouping data from different sources by density, isolation and combination of stations from different networks.

Five different estimation techniques were used in order to determine the best method and the best way to group data from different sources and the method that provided the best results was then chosen. Four of these methods were interpolation techniques which were applied and the fifth method is the GHI estimation based on satellite images (Meteosat) from the HelioClim3 Database [9] which is developed using the HelioSat2 method [10]. The four interpolation techniques used are: (Inverse Distance Weighting (IDW), Ordinary Kriging (OK), and two forms of Regression Kriging (RK)), the first technique is deterministic (IDW), and the rest are geo-statistic (OK, RK). The two forms of RK use three different auxiliary variables in order to improve their estimates: (i) GHI estimated by satellite; (ii) distance of location and current time of each observation compared to solar noon; and (iii) the geographical latitude. To account for the study's wide geographical scope, the five aforementioned GHI estimates were applied to six different ways of grouping the information available from the stations.

The best method for estimating GHI was defined based on the Relative Root Mean Square Error (%RMSE) of the difference between observed and estimated values; once the margin of error of the best method was determined, the authors proceeded to validate the volunteer network stations, labeling those whose error levels fell within the reference method.

Comparing the various spatial estimation techniques in the different ways of grouping results, made it possible to answer the following research questions:

(i) Is it possible, in mainland Spain and the Balearic Islands, to generate GHI surfaces with a 15-minute periodicity using GHI sensor observations from AEMet and CYL weather station networks with less error than satellite GHI estimates?
(ii) Do GHI values from the Meteoclimatic volunteer weather station network fall within the margin of error of official stations so that they may be considered valid?

In order to help answer this last question, a simple practical application of the research was carried out as a first approach for proposing to validate and include Volunteer Weather Observation (VWO) stations as an auxiliary source of data. This is in order to increase the density both in number and location of GHI sensors installed in a given region. This proposal is supported by the fact that the Internet has now made it possible for other parties aside from official agencies to publish weather data, allowing weather enthusiasts to quickly and voluntarily share observations from their stations, thus significantly increasing the amount of data available on this platform [11–13]. In this context, end-users play a significant role in producing information. This means that official data agencies are no longer the only parties producing information whether geographical, meteorological, *etc*. [13,14]. This is possible thanks to: (1) the declining price of weather stations, which makes it possible for private amateurs to acquire their own stations [15]; and (2) the emergence of a type of technology known as the Internet of Things (IoT) which makes it easier to share information from different types of devices or sensors, and particularly from weather stations, over global networks [16–18].

The movement that shares geo-referenced data is known as Volunteer Geographic Information (VGI). According to [19], VGI emerged as a form of Web 2.0. WikiMapia [20] and OpenStreetMap [21] are good examples of VGI [22]. Over the past few years, several studies have been undertaken in order to assess the quality of VGI data [22–26], a fact that points to a latent interest in the usefulness of this type of information. This movement is also active in the field of meteorology, in which volunteers assign a location and other metadata to their stations, albeit with the added particularity that in VWO surface stations automatically record weather observations, whereas this does not occur with the VGI movement but is rather part of what is known as passive crowd sourcing [27,28].

The aim of the present research project is to identify the best method for estimating 15-minute GHI in Spain based on interpolations applied to observations from two official station networks and contemplating the best way to group stations in the study area. A practical application of the research objective has been to compare GHI observations made by volunteer stations (Meteoclimatic) with the estimation model applied; this was done in order to identify those within the margin of error (average of ±2 standard deviations).

The remainder of article is structured as follow: Section 2, “Study Area and Source of Data” describes the geographical area in which this study is applied and also gives general information of the Data Sources. Section 3, “Methodology” describes the steps used to gather and grouping all data from different characteristics. The interpolation methods used and the validation criteria applied to the WVO stations. Section 4, “Results and Discussion” presents the results of all the prediction methods and provides an analysis of the winning ones and relates them to previous studies. Then, in this section is shown how these selected methods are applied for the validation of the VWO stations and the percentages of VWO labeled as possibly valid. The “Conclusion” section 5 presents key findings. The article ends with a section on “Future Works”.

## 2. Study Area and Source of Data

### 2.1. Study Area

The geographic area encompassed by the current study includes mainland Spain and the Balearic archipelago. It comprises an area covering 497,167 km^2^ (492,175 + 4,992) km^2^, and includes 16 of the 17 Spanish regions, and excludes the Canary Islands and the autonomous cities of Ceuta and Melilla located in northern Africa.

**Figure 1.**
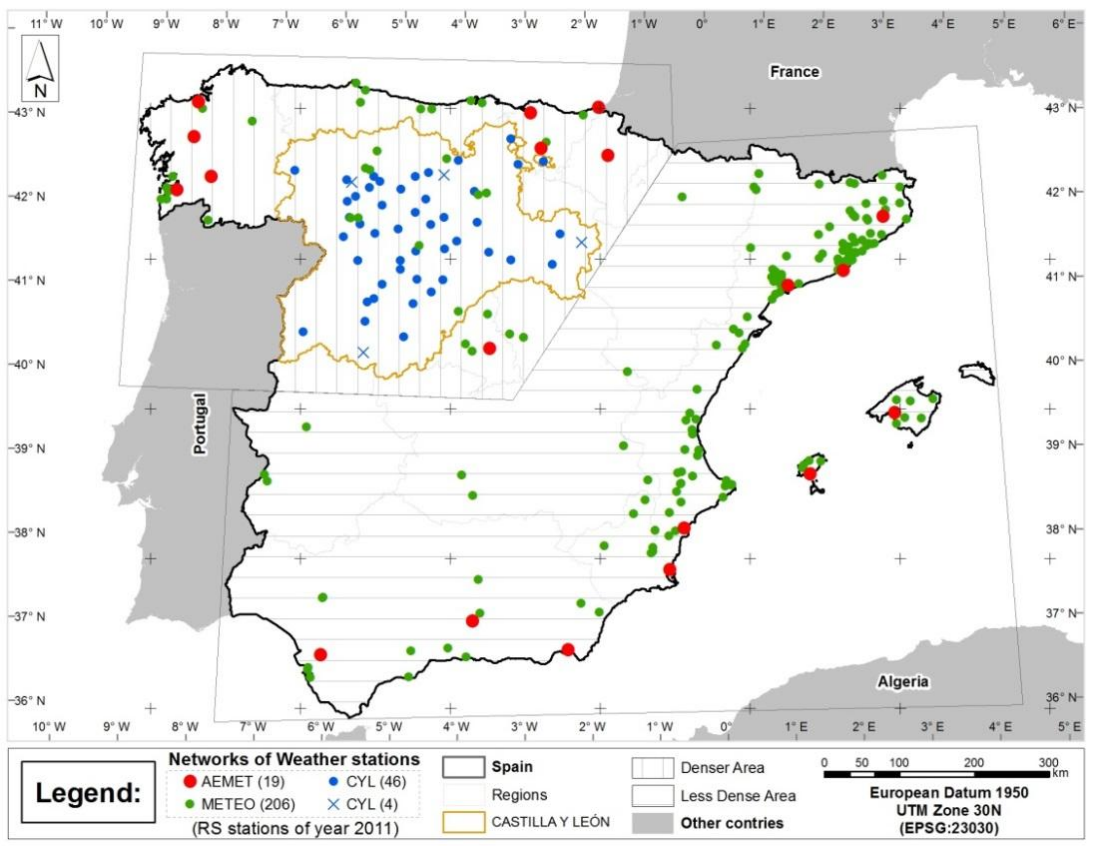
Distribution of weather stations.

Figure 1 shows a map of the study area with the distribution of the 211 weather stations from the three surface station networks involved. The lines on the grid are 200 km apart. The red dots represent GHI stations from Spain's official state meteorological agency (AEMet) and are distributed throughout the entire territory, albeit with a very low density. The blue symbols (dots and crosses) represent stations from the CASTILLA y LEÓN (CYL) network. This network is noticeably denser than the AEMet network, although its stations are limited to the region of Castile and León. Finally, the green dots represent stations from the Meteoclimatic (METEO) volunteer network located within the study area. Its stations are located mainly on the Mediterranean coast and in major cities [29].

The map also shows three interesting distinctions as far as density and the type of stations grouped. These sub-areas will be used in subsequent analytical processes and are described in Section 3.3. The sub-areas are represented by horizontal and vertical lines, and by the region of Castile and León. The first two sub-areas are artificial divisions. The third area corresponds to Spain's political division and delimits the region of Castile and León.

### 2.2. Data Sources

Four different data sources have been used; three are surface weather stations that recorded GHI observations with sub-hour periodicity in the year 2011 (a total of 206 stations) and the last source is the HelioClim3v2 data base of surface GHI estimates based on MeteoSat satellite images. A brief description of each source follows.

#### 2.2.1. AEMet

The Spanish National Meteorological Agency (AEMet), made the information from its weather stations openly available to citizens over the Internet for 2 years (from November 2010 through October 2012). During this period, the authors downloaded information from 23 stations that recorded GHI values in ten-minute intervals. For the purposes of this study, we have used only 2011 data from 19 stations (excluding four stations located on the Canary Islands) that have passed AEMet filters.

AEMet did a basic check of near real-time GHI observations [30]. Criteria for this basic control included: global SR no greater than a percentage of the extraterrestrial constant; diffuse SR no greater than global SR; global SR = diffuse SR + direct beam SR (on the same plane), and so on. Radiometers were also checked and calibrated attending to OMM standards [30].

#### 2.2.2. CASTILLA y LEÓN (CYL)

This network of automatic weather stations belongs the regional government of Castile and León which openly offers observations from its weather stations over the Internet [31] through the Castile and León Institute of Agricultural Technology [32]. The study contemplates 807,530 observations from the 50 stations in the network that recorded GHI values in 2011 with observations in 30-minute periodicity. The SR sensors in this network are calibrated once a year. The observations generated by this network are checked regularly using UNE standard 500504:2004 [33] and the seven quality control levels indicated therein [34].

#### 2.2.3. Meteoclimatic

As of June 2013, the Meteoclimatic (METEO) volunteer station network reported a total of 1,921 registered stations throughout the Iberian Peninsula, a fact that indicates the importance of this volunteer network. The network offers information from its weather stations over the Internet on websites [35] and through RSS subscriptions [36]. The present study uses the 4,581,643 Meteoclimatic observations from a total of 206 stations that recorded GHI observations for the year 2011 in Spain.

Quality control in this station network is non-standard and is not compulsory. The network's administrator along with its users bestow a seal of quality based on their experience and on a series of basic suggestions on how to install the sensors [37], they also bear in mind random errors found by users themselves [38]. At present, Meteoclimatic reports that 34.8% of its stations have followed this process.

#### 2.2.4. HelioClim3v2

The present study also uses GHI estimates derived from satellite images from version 2 of the HelioClim3 Surface GHI data base (HC3v2) [39]. This data base is created with HelioSat2 method [10], which uses satellite images taken every 15 min by Meteosat Second Generation (MSG) to estimate Solar Radiation on the Earth's surface [9,40]. HC3v2 GHI estimates were obtained via the Internet through the Solar Data Base Intelligent System (Soda-is) [41] web service.

Quality control for this data base has been carried out through 29 surface stations. GHI uncertainty was estimated following the benchmarking process recommended in Task 36 of the International Energy Agency's Solar Heating and Cooling Program (SHC) [42], this benchmark defines the use of the following thresholds (0.1; 10; 50 and 200) W/m^2^, resulting in mean square errors of 22.9%, 22%, 20.4% and 16.3% respectively [43].

## 3. Methodology

### 3.1. Data Collection and Temporal Adaptation

GHI information (observations) was downloaded periodically from the various sources (AEMet, CYL, METEO and HC3v2) using web-bots. These web-bots were developed according to the particularities of each of the sources to account for access protocols, data format, periods and latency of delivery for each networks as described in [29]. All observations were converted to the same time frames: UTC time and GHI values in minutes that are multiples of 15 (minutes: 0, 15, 30 and 45). AEMet and HC3v2 record their observations in UTC time, so no conversion was necessary, while METEO estimates UTC time by subtracting 1 or 2 h from the local time in Spain (depending on the time of year, winter/summer) and CYL estimates it by adding the local meridian offset with respect to the UTC meridian at each station's location.

GHI values at minutes that are multiples of 15 were estimated by linear interpolation of consecutive observations, recording the distance in minutes between such neighbors as an additional attribute. This time difference is subsequently used in the processes in order to identify observations, so that only observed values and interpolated GHI values based on neighboring observations not more than 30 min apart are taken into account. No conversion was necessary for HC3v2 since its estimates are offered in minutes that are multiples of 15. But, in CYL's case, the time at each station has a time reference corresponding to its local mean time, which results in shifting the original observations a few minutes when converting them to UTC time, such characteristics implies that most of this network's observations were interpolated.

Most of METEO's observations were also temporal interpolated for minutes that are multiples of 15, in this case, due to inconsistencies in the ways owners configure observation periods for their stations. As AEMet's observations are recorded every 10 min, only minutes 15 and 45 were interpolated based on observations recorded at minutes 10 and 20, and 40 and 50, respectively.

### 3.2. Preparing Explanatory Variables

According to Alsamamra *et al.* [44] explanatory variables must provide additional information for the Regression Kriging (RK) method and, therefore, their selection is a critical factor [44]. In addition, their correct use can compensate for the scarce geographic densification and distribution of the variable to be interpolated. These variables are applied in RK methods used in the present studies in Sections 3.4.4 and 3.4.5.

Various factors can influence the GHI levels perceived in a given position on the Earth's surface. Different studies take different factors into account, so [44] uses variables derived from the Digital Elevation Model in order to estimate average monthly GHI; Antonanzas-Torres, Cañizares and Perpiñán [8] estimates annual irradiance values for three different planes using irradiance values at different angles on the planes as auxiliary input variables; Evrendilek and Ertekin [45] estimated average monthly SR using a wide range of auxiliary variables: latitude, longitude, altitude, aspect, distance to the sea, minimum and maximum temperatures, relative humidity, soil temperature, cloudiness, precipitation, evapotranspiration, length of day, sunshine duration, average daily solar radiation, month of the year [45]; while Ertekin and Evrendilek [46] used only sunshine duration as an auxiliary variable in comparing 18 empirical models for estimating average monthly GHI; Moreno, Gilabert and Martínez [47] use minimum and maximum temperatures and precipitation to estimate daily GHI; Bojanowski, Vrieling and Skidmore [48] calibrated coefficients for three models for daily SR using sunshine duration, cloudiness and air temperature as auxiliary variables; Alsamamra *et al.* [44] and Kumar, Skidmore and Knowles [49] modeled the topographical variation of SR using the weather station that determines the sun's angle, atmospheric conditions and shadows made by the varying heights of the terrain as auxiliary variables.

Previous studies have not focused on 15-minute GHI values. Three auxiliary variables were selected in the current study in order to contribute additional information about changes in GHI in 15-minute periods to the interpolation methods used.

The first explanatory variable is an abstraction of the continuous change in the sun's angle which makes the sun appears to move from day to day throughout the year and throughout each day. It is structured as a standardized distance calculated for each GHI observation from its True Solar Time (TST) at Solar Noon Time (SNT) with respect to dawn and dusk at each station for each day in question. This distance has no units and its values are between −1 and 1, where the value of −1 is the moment at which TST is equivalent to dawn, the value of 0 (zero) occurs when TST is equivalent to SNT (no distance, and the ideal time for greater GHI), and 1 is when TST is equivalent to dusk. D_SNT values outside these ranges imply that the sun is above the horizon and therefore the stations have GHI values equivalent to zero. This is also important as a filter, because allows us to reduce the number of observations to be processed by approximately a third (corresponding to nighttime periods).

Therefore this variable, labeled D_SNT, was created independently of the location of each station, date and time. The calculus of D_SNT implied contemplating a total of eight components that must be determined and derived beforehand; Two of these are existing variables: (i) UTC Time for each observation; and (ii) the longitude of each station's location, these two variables are in turn used as input processing data for the following components: (iii) dawn; (iv) dusk; (v) TST; (vi) SNT; (vii) decimal time; and (viii) time equation. The astronomical formulas for these last components are listed in [50]. D_SNT is one of the explanatory variables used in the Regression Kriging method in Section 3.4.4.

The second variable is composed of GHI estimates from remote sensors (E_GHI). This variable was selected following the trend set by other studies that use a version derived from satellite images of the target variable as an auxiliary variable. For instance, Hengl *et al.* [51] used MODIS for temperatures. In our study, E_GHI is obtained directly from the HC3v2 data base. Each E_GHI value is associated with its corresponding observation of surface GHI (AEMet, CYL and METEO). In addition to using E_GHI as an auxiliary variable for the fourth spatial interpolation method (Section 3.4.5), it was also used to compare satellite-based SR estimates and observations of surface SR.

The third variable is the geographical latitude. This variable was obtained directly from the location for each station. This third variable were used over the two RK methods (Sections 3.4.4 and 3.4.5) and it was selected on base on the known fact that for clear sky conditions, places closer to the Equator have greater SR values independently of the season, day or time [49], for instance, on [45] was found a global South-North trend for Turkey.

**Figure 2.**
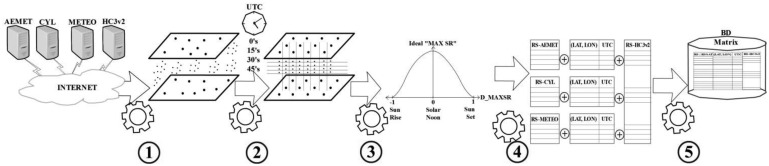
Preparing and collecting data.

Figure 2 is a graphic representation of each of the stages of the method described so far (1, 2, 3 and 4). It also reflects the last stage (5) which represents the input data matrix for the next steps of the methodology, consisting of the information that was collected and processed: GHI, E_GHI, D_SNT, TST, Dawn, Dusk, UTC time, type of GHI value (observed/interpolated), station location (longitude, latitude) and additional information about the station (Station ID, Station Name, Origin, *etc.*). In this figure, the various stages are represented using a gear metaphor. Each stage has one or more inputs, located to the left. The first data sources are Internet servers and the last source consists of a data base with the resulting matrix up to this point. The output for Process 1 illustrates a set of somewhat disorganized points, representing the data sources' various types of local times and several kinds of observation periodicities. Process 2 takes the information above and makes it coincide in a single time frame. Process 3 illustrates calculations for the first auxiliary variable (D_SNT). Process 4 represents the association between surface GHI observations and their respective estimates via satellite E_GHI (second auxiliary variable) based on UTC Time and on the stations' locations.

### 3.3. Grouping Source Data

**Figure 3.**
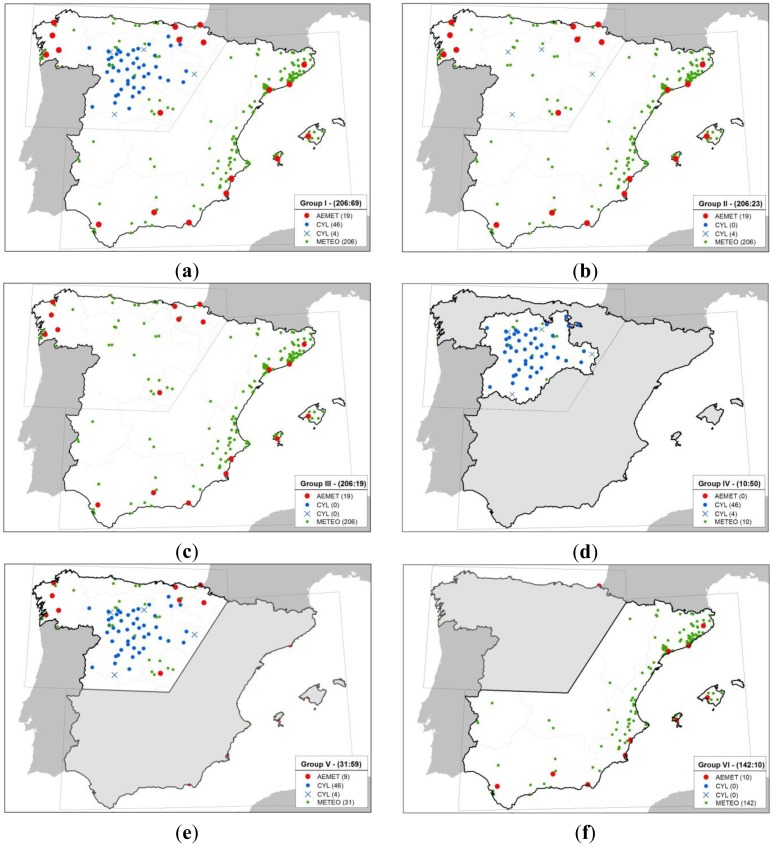
Six different ways to group the data to be analyzed. (**a**) Group I (AEMet + CYL); **(b)** Group II (AEMet + 4 CYL); (**c**) Group III (AEMet); (**d**) Group IV (CYL); (**e**) Group V (CYL + 9 AEMet); (**f**) Group VI (10 AEMET).

The analysis was carried out by grouping data in six different geographical dataset so as to account for the effects of zones with greater or lesser station density. Figure 3 illustrates the various groupings in six maps. Figure 3a–c contemplates the use of the total study area in order to subsequently validate all the volunteer stations contained. These three groupings differ in the way stations are combined. Only Group I (AEMet + CYL), Figure 3a combines all the official stations in the study area in order to generate spatial estimates with which to subsequently validate volunteer stations. The ratio of volunteer to official stations is 2.98:1.

Group II (AEMet + 4 CYL), Figure 3b uses two official sources, including all AEMet stations and 4 CYL stations that are approximately equidistant from each other. Four of the 50 stations were selected to break up the cluster generated by all the CYL region stations in the study area. This election penalizes the proportion of volunteer stations compared to official stations by about 8.95:1.

Figure 3c presents Group III (AEMet), which includes only AEMet stations for the entire study area as the network is evenly distributed throughout the area. In this case the ratio (volunteer:official) is 10.84:1.

Figure 3d,e,f represents groupings that include sub-areas of the study area and includes only stations that fall within said sub-areas irrespectively of the source. Figure 3d represents Group IV (CYL), which includes only the CYL region and does not include any AEMet stations. Its ratio favors official stations by 1:5 (volunteer:official).

Group V (CYL + 9 AEMet) is illustrated in Figure 3e and includes the northeastern area of the Spanish mainland. This area presents a ratio of 1:1.9 (volunteer: official). Conversely, Figure 3f shows Group VI (10 AEMET) and includes the area with the least number of official stations, with only 10 AEMet stations; it is also the area with the most volunteer stations, with a disproportionate ratio of 14:1 (volunteer:official).

### 3.4. 15-Minute Spatial GHI Estimates

The following processes were carried out independently for each of the six groupings of data explained in Section 3.3. Spatial prediction was carried out by grouping GHI observations in 15-minute time periods and processing them independently, thus avoiding having to account for GHI variability throughout the day and throughout the year. All procedures and calculations in the methodology from this point on were carried out using R software [52]. Interpolation processes use the R *gstat* geostatistics package [53], with the sole exception of generating semivariograms (see Section 3.4.3), for which R *automap* package is used [54]. All the interpolation methods used in this study are based on 2D Cartesians coordinates without taking into account the increment in altitude for the distance calculations.

#### 3.4.1. Detecting Outliers

Outliers were detected using BoxPlot [55] based on deviations between observations and estimates, labeled (Y) and (Ŷ), in the various analytical processes. BoxPlot organizes the results of deviations in order to obtain the first (Q1) and third quartile (Q3) of said values, and uses them to calculate the inter-quartile value (IQ = Q3 − Q1). It then defines the lower and upper limits of values considered minor outliers (those that are outside these limits) and valid values (those within the limits). Left of Q1, these limits were established at −1.5 of the value IQ, whereas to the right of Q3, they were set at 1.5 of the IQ.In order to prevent observations from these official stations to add noise to the interpolation processes, despite having passed systematic controls, it was first necessary to detect those stations that behaved abnormally throughout the day in order to leave them out in future processing for each day in question. In this case, observations (Y) are the 15-minute GHI values and estimates (Ŷ)are obtained by satellite (E_GHI). Their deviations were grouped by day and station and added using two statistics:

(i) Root Mean Square Error (RMSE) (Equation (1)); and (ii) Mean Absolute Error (MAE) (Equation (2)), to which we applied BoxPlot's outlier detection:

(1) $$\text{RMSE}=\sum_{i=1}^{n}\sqrt{\frac{1}{n}{\left(Y_{i}-{\hat{Y}}_{i}\right)}^{2}}$$

(2) $$\text{MAE}=\frac{1}{n}\sum_{i=1}^{n}|Y_{i}-{\hat{Y}}_{i}|$$

Finally, stations identified through this means were considered outliers, whether they were detected through RMSE or MAE. Having identified the valid official stations for each day, the authors then proceeded to use those stations' observations in the four spatial interpolation methods described in the following sub-sections.

#### 3.4.2. Method 1: Inverse Distance Weighting (IDW)

In this method, the influence between the nearest and farthest observations from the point to be interpolated is deterministically/analytically defined. The weights assigned to each station's observations are inversely dependent on the distance between the station to be interpolated and the reference stations. Since only the relative proximity of observations to the point to be interpolated was taken into account, the smaller the distance, the greater the influence of GHI values observed by the stations. Various studies use IDW to interpolate SR [56,57]. The general formula for IDW interpolation is presented in Equation (3):

(3) $${\hat{z}}_{idw(s_{0})}=\frac{\sum_{i}^{n}\frac{Z_{i}}{d_{i0}^{\beta }}}{\sum_{i}^{n}\frac{1}{d_{i0}^{\beta }}}$$

where *ẑ*_*idw*(*s*_0_) is the estimated value of the node *s*_0_; *n*, is the number of points observed; *Z_i_*, the value of observation i, and *d_i_*_0_ is the distance from point *i* to the node *s*_0_ to be estimated. Both the main denominator and weighting value *β* verify that weight diminishes with distance. For this particular study, the term *β* is equal to 2, which is the most popular choice and the default value proposed by *gstat*.

#### 3.4.3. Method 2: Ordinary Kriging (OK)

Both the (OK) method, and the next two methods outlined in Sections 3.4.4 and 3.4.5 are geostatistical methods. Geostatistics models a semivariogram, which accounts for both the distance between the points observed and the differences in their values in order to objectively define the weights to be used in interpolations. The OK method has been used on various occasions in order to estimate SR [48]. Equation (4) presents the general OK formula, whereas Equation (5) presents the formula for the experimental semivariogram. Where ẑ_ok(s_0_) is the estimated value for node s_0_; n is the number of observed points used; w_i_ is the Krigrings weight vector, and Z_i_ is the value of observation i:

(4) $${\hat{z}}_{ok(s_{0})}=\sum_{i=1}^{n}w_{i}(s_{0})z(s_{i})$$

(5) $$\gamma (h)=\frac{1}{2}\sum [{\left(z(s_{i})-(z(s_{i}+h)\right)}^{2}]$$

Here, *z*(*s_i_*)is the value of the observation at a given point, *s_i_*, and *z*(*s_i_* + *h*) is the value of the neighboring observation located at a distance *h*. This is how the various observations are assigned weights with respect to the node to be interpolated. Once the experimental semivariogram has been processed, the authors proceeded to calculate the variogram model which can be: linear, spherical, exponential, circular, Gaussian, Bessel, power or the like [58]. The R *automap* [54] package was used both to calculate the experimental semivariogram and process and select the best-suited semivariogram model.

#### 3.4.4. Method 3: Regression Kriging with D_SNT and Latitude as Auxiliary Variables (RK1)

Regression Kriging (RK) is a geostatistical method that includes information aside from the variable to be interpolated (in the form of explanatory auxiliary variables) in order to improve the interpolation process with information that is better distributed spatially. Auxiliary variables have a more suitable spatial dispersion and thus serve as proxies for the target variable, thus compensating the target variable's relatively scarce spatial distribution [44,48]. As in OK, RK is based on the variogram model used to generate interpolations; In addition, RK is composed of the sum of the stochastic and determinist parts of the spatial variation of the target, both of which may be modeled independently and processed in two steps, as demonstrated in Equations (6) and (7). In the current study, the authors first found the deterministic part by establishing Multiple Regression Linear Models (MRLM) between the GHI and the auxiliary variables. Then the stochastic part was carried out, which consisted of executing the OK for the residuals of the MRLM. The sum of both values generates the final GHI estimate at a given point. The RK method has been used on various different occasions to estimate SR [8,44–46], and also to interpolate other weather and climate variables [51,58–60]:

(6) $$\hat{z}(s_{0})=\hat{m}(s_{0})+\hat{\varepsilon }(s_{0})$$

The target variable for the estimate is a function of the sum of the deterministic and stochastic models, first and second terms of the equation respectively. The parts of Equation (6) are derived in Equation (7):

(7) $$\hat{z}(s_{0})=\sum_{k=0}^{p}{\hat{\beta }}_{k}q_{k}(s_{0})+\sum_{i=1}^{n}\lambda_{i}\epsilon (s_{0})$$

In the expression, *p*, is the number of auxiliary variables; *β̂_k_*, is the MLRM estimate of coefficients; *q_k_*(*s*_0_), are the auxiliary variables at the given *s*_0_ points; *λ_i_*, are the Kriging weights determined by the spatial dependency structure of the residuals, and y *ϵ* (*s*_0_), are the residuals at the given *s*_0_ points. A distinctive feature of the RK1 method is that the input auxiliary variables are D_SNT and the latitude of the station's location.

#### 3.4.5. Method 4: Regression Kriging with E_GHI and Latitude as Auxiliary Variables (RK2)

The RK2 method uses the same system of equations and processes explained in the previous section for RK1 only with different auxiliary variables. Here, the auxiliary variables are E_GHI, and the station's latitude. Both RK methods use latitude as the second auxiliary variable. However, E_GHI and D_SNT are not used together in any RK method. This is because both these variables present near-extreme multicollinearity. When all *ante meridiem* “a.m.” (D_SNT ≤ 0) and *post meridiem* “p.m.” (D_SNT > 0) observations are separated, the a.m. correlation value of both variables is 0.7825 with a coefficient of determination R^2^ of 0.6124, and its p.m. correlation value is −0.8102 with R^2^ of 0.6566. Since both variables are conceptually different and have R^2^ > 0.6, the decision was made not to use them together so as to avoid making the MLRM more sensitive to minor errors [42] (pp. 141, 150).

### 3.5. Validation of the Methods

Leave-one-out cross-validations were carried out in order to determine the precision of each model and verify which of the four methods of interpolation was most suitable. This was done using data from all the stations except one, which was hidden in order to then estimate the value at its location and compare the result to the observed value. The precision of the HelioSat2 model was obtained based on the direct estimates in the HC3v2 data base, calculating the differences between surface observations and estimates. In all cases, relative RMSE (%RMSE) was calculated based on the ratio between statistical error and the average observed variable multiplied by 100, as may be observed in Equation (8):

(8) $$\%\text{RMES}=\frac{\text{RMES}}{\frac{1}{n}\sum_{i=1}^{n}\left(Y_{i}\right)}\times 100$$

This %*RMSE* indicator was calculated independently for each of the six ways of grouping data and for each set of 15-minute observations, which were then processed and grouped by day. In order to compare results from several models previously mentioned with the reference benchmark in [43], the authors calculated errors for 5 GHI thresholds (NA; 0.1; 10; 50 and 200) W/m^2^, which were also used by [43] to calculate uncertainty in the HelioSat2 model. By applying no threshold (NA) and applying the threshold (0.1), it was possible to discern the contribution of very small outlying GHI values <0.1, while other thresholds helped gauge how the various estimation methods responded to eliminating low GHI values.

### 3.6. A First Approach to Validating Volunteer Stations

After finding the best combination of estimation method and way of grouping data as indicated by the best error value and its standard deviation (std-dev), said combination was chosen to estimate GHI values at volunteer station sites. The authors then proceeded to classify the volunteer stations' GHI observations according to whether they fell within or outside that error range and these classifications were finally quantified by day and by station in order to determine which stations behaved in “valid” and “invalid” ways as far as errors in the interpolation models or satellite estimates.

**Figure 4.**
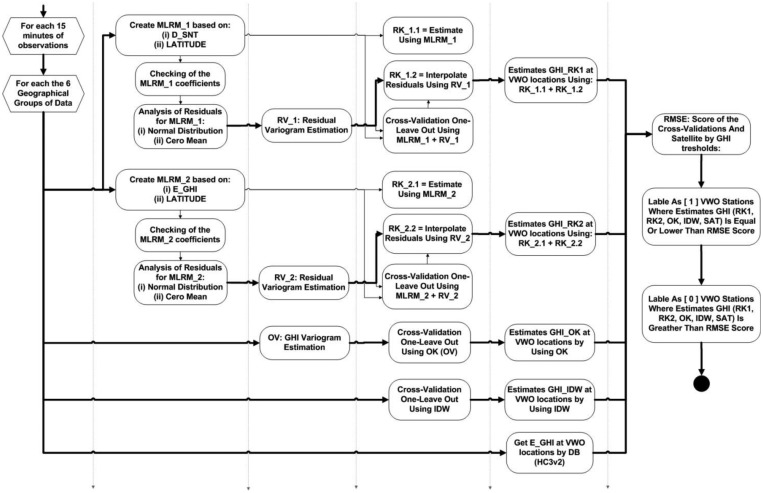
Work flow of the method's analytical processes.

Figure 4 represents the work flow which in summary includes: for each 15-minute of observations, for each one the six different groupings of observations, cross-validating methods of interpolation, interpolating the best method/grouping at the volunteer station locations, and classifying of the volunteers found within the margin of error for the estimates.

## 4. Results and Discussion

### 4.1. Scoring the Methods of Estimation

The precision and scoring of methods of estimation in each of the geographical groupings was obtained by calculating daily aggregations of %RMSE based on individual 15-minute differences (GHI-E_GHI) and eliminating days identified as atypical using the BoxPlot method (see Section 3.4.1). These scores are presented in Tables 1 and 2; Table 1 presents the statistics without establishing thresholds for the values recorded in GHI observations, while Table 2 presents the statistics and establishes the threshold for SR at values greater than 200. This threshold prevented us from verifying the effect of estimating RS when values are low (Table 1). When said values are excluded (RS ≤ 200), the various methods of estimation produce better results.

The values in both tables indicate that the RK2 and SAT methods always win, no matter the type of statistic or threshold used. The SAT method always wins using Groups (III and VI), and RK2 always wins with Groups (I, IV and V). When all observations are taken into account, the winner for Group II is RK2. However, when only GHI observations greater than 200 are considered, the winning method is SAT, which suggests that the SAT method improves with greater GHI values.

Groups (III and VI), in which SAT is the winner, only include AEMet stations, so they have the least number of stations per work area and therefore lower station density (see Table 3). It may also be observed that, every time the SAT method wins, the second best method is RK2.

Groups (I, IV and V), in which RK2 is the winner, are characterized by the fact that they contain all the CYL stations in addition to other AEMet stations. This implies a greater number of stations and therefore greater density. Here, although method RK2 uses satellite estimates as an auxiliary variable, Groups (IV and V) present SAT as the best method. Groups (IV and V) have greater station density, which means that, on average, the stations are closer to each other (the average distance between stations is lower), as may be observed in Table 3. In these cases, the second best method is RK1, except in Group V without the limitation of a threshold. In all the other groups, the second best methods were RK2 and SAT.

Bearing in mind the possible relationship between the winning methods and the number of stations in each type of group, as well as the average distance between neighboring stations, Table 3 presents the statistics for each of the groups. It is possible to observe that those groups that include all the CYL stations (IV and V) present more suitable statistics (lower average distance between neighboring stations). The opposite is true for groups that only contain AEMet stations (III and VI), which cover areas with fewer stations.

The best method selected for each group was the one that presented the lowest average aggregate value. Tables 4 and 5 present these winning methods along with other relevant values (average, standard deviation, percentage of days ruled out because the method presented atypical estimates when applied to them, percentage of days on which the method obtained better average values than the other methods in that same group). Table 4 shows those cases in which all GHI values were taken into account, while Table 5 only shows cases with a GHI threshold >200.

In both Tables 4 and 5 it may be observed that applying method RK2 to Group IV (CYL) resulted in the lowest (best) average value, however, the present research project cannot select this method/group as the only winner because it does not encompass the entire study area. Here too, we see that Group VI (10 AEMet) combined with method SAT has the second lowest average value, but this group doesn't cover the entire study area either. Given that even the combined areas of the two best groups/methods (IV and V) do not cover the entire study area; the study has also taken into account a third group/method. In this case, the authors chose Group (V) combined with method RK2. This third group/method presented the next best average value, and its extension also includes part of the remaining area of interest. Another characteristic worth highlighting about the three groups chosen is that, with a threshold of GHI > 200, their standard deviation values are between 6.24 and 7.96%. These values are not very far from the maximum 5% considered acceptable for properly maintained stations with hourly observations according to [56]. It is plausible that the difference presented in this study is due to working with intra-hour observations. On the other hand, all three of the remaining groups that were not selected (I, II and III) and the methods applied contemplate the entire study area, yet all three also present higher average values.

### 4.2. Detailed Analysis of the Winning Methods

**Figure 5.**
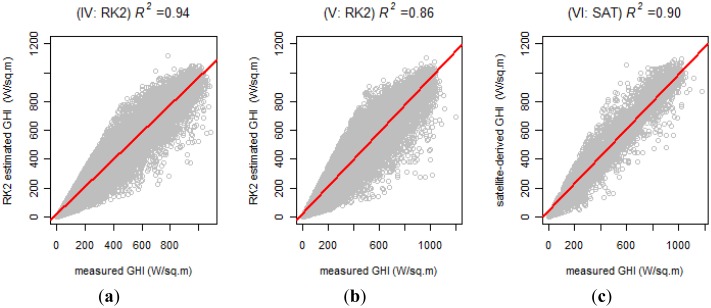
Relationship between estimated GHI and GHI observed by surface stations: (**a**) RK2: Group IV (CYL); (**b**) RK2: Group V (CYL + 9 AEMet); (**c**) SAT: Group VI (10 AEMet).

Having defined the combination of the three groups/methods that cover the study area, we proceeded to analyze each of these. Figure 5 presents the scatter plots, regression line, and correlation coefficients (*R*) and the coefficients of determination (*R^2^*) for each of these. For instance, in Figure 5, the clouds of points only show 5% of the values selected at random from non-outlying measured/estimated values (as per the BoxPlot method of estimating differences). The values of the coefficients and the regression line are based on the entire universe, without discriminating outliers. The three winners in their respective groups present a strong correlation, with *R* values of 0.97; 0.92 and 0.95. In addition, Figure 5 shows high *R^2^* values for the three winners: 0.94; 0.86 and 0.90.

Next, we proceed to analyze certain remarkable characteristics and limitations in each of the winning methods.

#### 4.2.1. RK2 in Group IV (CYL)

Table 6 presents the statistics for the RK2 method in Group (IV-CYL) for 6 thresholds (all, 0.1, 10, 50, 200) W/m^2^. Here is shown that percentage statistics (%RMSE and %Std-Dev.) decrease as GHI thresholds are set, from an average of 22.46 and std-dev of 2.87 for the (0.1) threshold, to approximately half of those values (11.26 and 1.35 respectively) for the (200) threshold. However, absolute statistics present a slightly upward trend: (68.95 and 10.36 W/m^2^) for the (0.1) threshold, and up to (72.21 and 12.97 W/m^2^) for the (200) threshold.

%RMSE and RMSE values in the table above are lower than the respective values (22.9, 20.2, 20.04, 16.3)% and (69, 72, 77, 83) W/m^2^ for the (0.1, 10, 50 and 200) W/m^2^ thresholds of the benchmark in [43].

It may also be observed that there is no difference between the (0.1) threshold and no threshold (NA) for statistics with no outliers. However, for those same thresholds, there is a significant drop in %RMSE (All) statistics and Std.Dev (All) of 40% (149.74/247.31) and 45% (362.46/508.06) respectively. The importance of this fact calls attention to how very small SR values (GHI < 0.1) contribute to outliers. It is also possible to observe that this trend continues, although to a lesser degree, with low threshold values (10 and 50). This demonstrates the method's limitation when estimating low GHI values.

**Figure 6.**
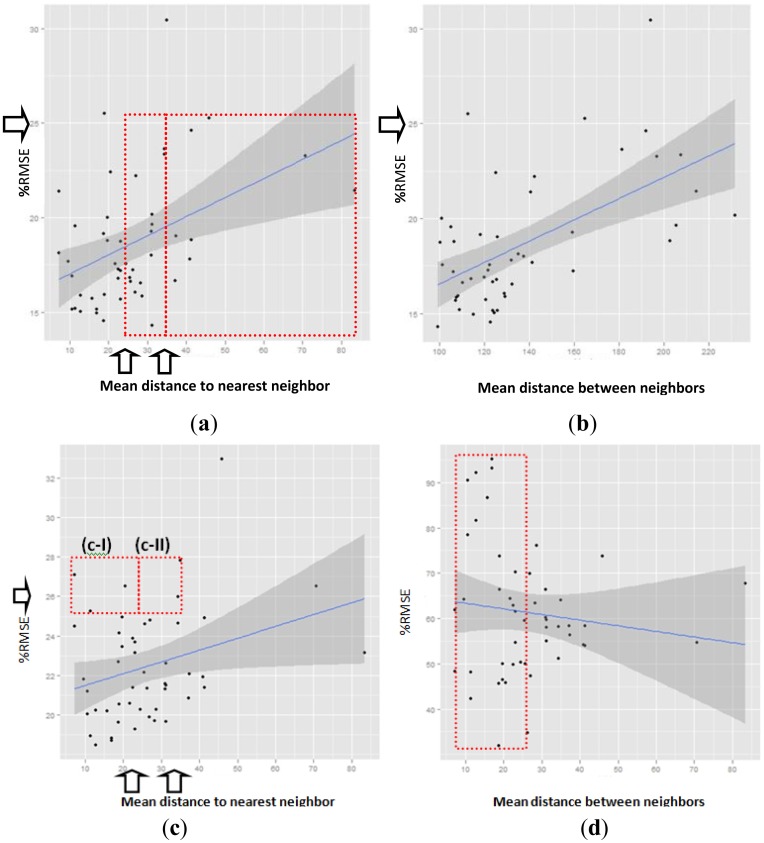
%RMSE compared to neighbors for methods RK2 in CYL. (**a**) %RMSE compared to the nearest neighbor for the 200 threshold; (**b**) %RMSE based on the average distance between neighbors for the 200 threshold; (**c**) %RMSE based on the nearest neighbor without established thresholds; (**d**) %RMSE of the Estimates derived by satellite based on the nearest neighbor without establishing thresholds.

**Figure 7.**
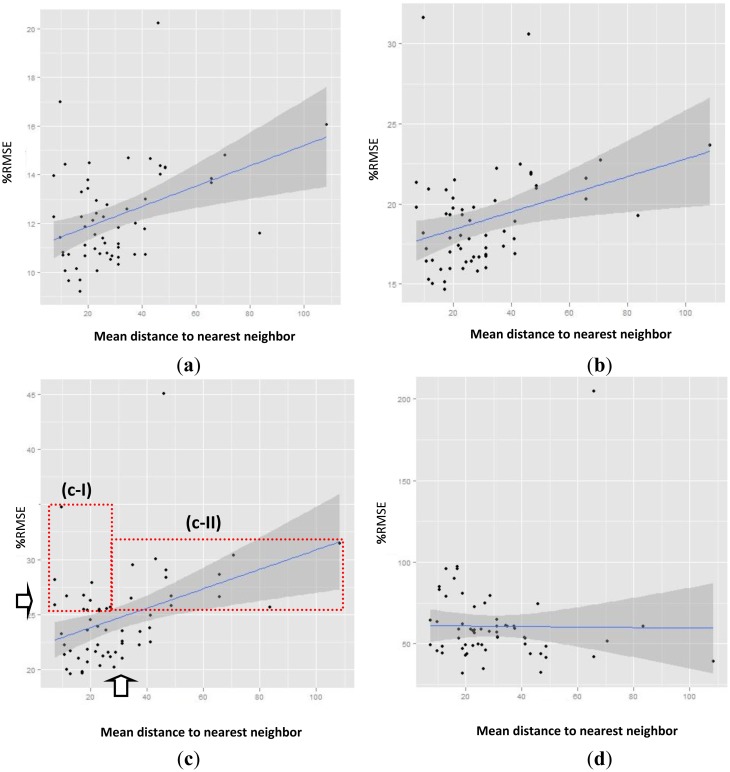
%RMSE compared to neighbors for method RK2 in Group VI (CYL + 9 AEMet). (**a**) %RMSE compared to nearest neighbor for the 200 threshold; (**b**) %RMSE compared to nearest neighbor for the 50 threshold; (**c**) %RMSE based on the nearest neighbor without established thresholds; (**d**) %RMSE of the Estimates derived by satellite based on the nearest neighbor without establishing thresholds.

Figure 6 presents %RMSE according to the distance separating the point to be interpolated from neighboring points. This proves the strength of the method applied herein and makes it possible to compare the results of the present study with those of [56,57]. These graphics, like those in Figure 7, include an estimated linear regression line (straight blue line) as well as its corresponding confidence interval (grey area). The arrows on the “Y” axis point to 25%RMSE, and the ones on the “X” axis point to the 25 and 34 Km limits indicated in the above-mentioned studies. Figure 6a shows %RMSE with a threshold >200. This chart shows a “nugget” effect between 15%–17%, similar to that reported by [56,57]. It suggests that the nugget effect is due to the discontinuous nature of the spatial structure for hourly GHI (*i.e.*, cloud-blue sky). It is worth pointing out that, despite this and other subsequent similarities, said studies used a variant of IDW as a method for interpolation. They also used hourly GHI data and GHI values with a RMSE range between (80–100) W/m^2^, while the RMSE range for 15-minute data used in the present research is between (62–88) W/m^2^. The main contribution that is shown in Figure 6a is that %RMSE is less than or equal to 25.5%, irrespectively of the distance separating the point to be interpolated from the nearest neighbor, and irrespectively of the average distance to all neighbors in Figure 6b.

This makes the RK2 method stand out, because, despite working with intra-hourly GHI observations, they do not exceed the limit of 25 %RMSE for hourly observations indicated by [56,57]. Said studies indicate that interpolations based on surface observations beyond 25 km (in the first study) and 34 km (in the second) exceed 25%RMSE and, at greater distances, satellite estimates are more precise and therefore their use is preferable. The red rectangles in Figure 6a represent stations that, according to the aforementioned studies, should have presented their %RMSE's greater than 25% in their interpolations. On the other hand, the results shown here also present another improvement compared to the intra-hourly observations presented in [56] where the “nugget” effect is 22% and from approximately 20 km %RMSE exceeds 30%, reaching approximately 40% at 80 km.

Figure 6c, as well as Figure 6a, presents the relationship between %RMSE for each station and its nearest neighbor. The difference between them is that there are no set GHI thresholds. This figure shows how including GHI values lower than 200 W/m^2^ increases the nugget effect up to 20%–22.5%. The red rectangles (c-I and c-II) also indicate that there are six stations that exceed the 25% threshold, although they do not reach 28%. However, five of these six stations are within 34 km and three of these five are within 25 km, so it is plausible to assume that exceeding the 25% threshold for %RMSE is a consequence of the low GHI values and not of the distance of the nearest neighbor. The only exception to all the above is seen in station (BU101) with a distance of more than 35 km from its nearest neighbor and a %RMSE of 35%.

Figure 6d illustrates how the SAT method behaves, with GHI estimates derived from satellite images (E_GHI). For Group IV, this method was found to be in last place in all cases (see Tables 1 and 2). As was to be expected, this figure shows that there is no correlation between the distance of the estimated points and %RMSE of the E_GHI; At first glance, it may be observed that there the points (inside the red box) are very dispersed along the entire “Y” axis between 7 and 20 km. Likewise, these %RMSE values are greater than those for the RK2 method in Figure 6c.

#### 4.2.2. RK2 in Group V (CYL + 9 AEMet)

Table 7 presents the statistics that result from applying the RK2 method to Group V (CYL + 9 AEMet) for 6 GHI thresholds (all, 0.1, 10, 50, 200) W/m^2^. %RMSE with a threshold of 50 and 200 (18.95 and 12.29)%, are better than the respective values of (20.04 and 16.3)% of the benchmark in [43]; while RMSE values with thresholds from 10 (70.49, 68.95 and 63.39) W/m^2^ are better than the equivalent values in said benchmark. In general, we find the same behavior as in the previous Section 4.2.1 and Table 6 with respect to the decrease of %RSME and its std-dev as threshold values increase. However, in this case, the relative averages and their standard deviations also decrease, unlike what occurs with (RK2 Group IV). Another difference worth highlighting with respect to the above-mentioned method is that, here, it always presents better values than the aforementioned benchmark for all thresholds.

Figure 7 presents the relationship between %RMSE and nearest neighbors. Figure 7a shows the relationship between %RMSE and nearest neighbor for the >200 threshold. In this case, the nugget effect is approximately 11%RMSE, and, in the entire study area, %RMSE remains, on average, below 20.22%, irrespectively of the distance from the nearest neighbor (which ranges from 7.3 km to 108.4 km). In addition, most of the points (except three points), are less than 15%RMSE. For the (GHI > 200) threshold, the relationship between %RMSE and average distance from neighboring stations follows a similar trend to that between %RMSE and distance from nearest neighbor, as was also the case in Figure 6b.

This is one of the reasons why here Figure 7b shows the relationship between %RMSE and nearest neighbor for the >50 threshold. The other reason is that all points (except two points) are on average less than 23.68% of the %RMSE irrespectively of the distance from the nearest neighbor. It is worth noting that, with thresholds of 50 and 200, the RK2 method combined with Group V (CYL + 9 AEMet) does not exceed the limit of 25%RMSE beyond 25 km or 34 km for hourly observations indicated by [56,57].

The above-mentioned values represent an improvement with respect to the method (RK2 Group IV) used in Section 4.2.1, proving the importance of setting up more stations in order to carry out the interpolations (59 stations for Group V, as opposed to 50 for Group IV, see Table 3).

Figures 6c and 7c present the relationship between RMSE for each station compared to its nearest neighbor without a set threshold. This figure shows how including GHI values lower than 200 W/m^2^ or lower than 50 W/m^2^ increases the nugget effect up to 21%–23%. This figure presents a large number of points (42% of them), contained between the red rectangles, which exceed the 25%RMSE threshold. Of these points, half (22%) of those shown in the rectangle (c-I) are found within 34 km, and, in turn, most of these (19%) are within 25 km. So that, as with Section 4.2.1 (RK2 in Group IV), it is plausible to assume that exceeding the 25%RMSE threshold is dependent on low GHI and not on the distance from nearest neighbor.

Figure 7d illustrates how the SAT method behaves, which are the GHI estimates derived via satellite (E_GHI) in this Group V. In this case, SAT is ranked as the last method for statistics without thresholds (see Tables 1 and 2). As expected, this figure shows that there is no relation between the distance between points and %RMSE for E_GHI; It also shows that these %RMSE values are greater than those for the RK2 method in Figure 7c.

#### 4.2.3. SAT in Group VI (10 AEMet)

Table 8 presents the statistics for the HelioSat2 method in Group (VI- 10 AEMet) for six thresholds (all, 0.1, 10, 50, 200) W/m^2^. As is also the case with the aforementioned RK2 methods, relative averages decrease as the threshold levels increase. However, with these estimates, standard deviations are not only lower, they also increase as threshold levels rise (contrary to what occurs in the RK2 methods of previous sections). Despite this, the highest std-dev value 1.27% is lower than the lowest value in the previous cases, 1.35% (in Section 4.2.1). With respect to the benchmark in [43], it is worth noting that although the %RMSE for the 0.1 and 10 thresholds are very similar, these same values for the 50 and 200 thresholds are lower than those for the aforementioned benchmark and similar to those in the previous RK2 cases in Sections 4.2.1 and 4.2.2.

Although the winning method for Group VI (10 AEMet) is SAT, it is to be expected that %RMSE for GHI estimates derived by satellite (E_GHI) will not depend on the distances between stations given that the E_GHI for the HelioSat2 method do not depend on surface observations. This also occurs with Groups IV and V in Sections 4.2.1 and 4.2.2 (see Figures 6d and 7d).

However, it is worth noting that E_GHI present similar %RMSE values in all thresholds compared to the RK2 method in Groups IV and V. The authors believe this is due to the fact that this group only includes stations from the AEMet network.

### 4.3. Analysis of First Approach for Volunteer Station Validations

The detailed analysis of the best method of estimation for the three Groups (IV, V and VI) in Section 4.2 allowed us to discount the method/Group IV in favor of the method/Group V. This is due to two reasons: (1) In Group V, the RK2 method improves its %RMSE ratio with respect to distance from nearest neighbor as it includes 9 AEMet stations; (2) Area V contains Group IV's area. Therefore, in order to validate volunteer stations in the entire study area, it is used as the best method/group, the combination of the RK2 method in Group V, and the SAT method in Group VI, as shown in Equation (9):

(9) Method/Group for the whole area=RK2(in Group V)∪SAT(in Group VI)

**Figure 8.**
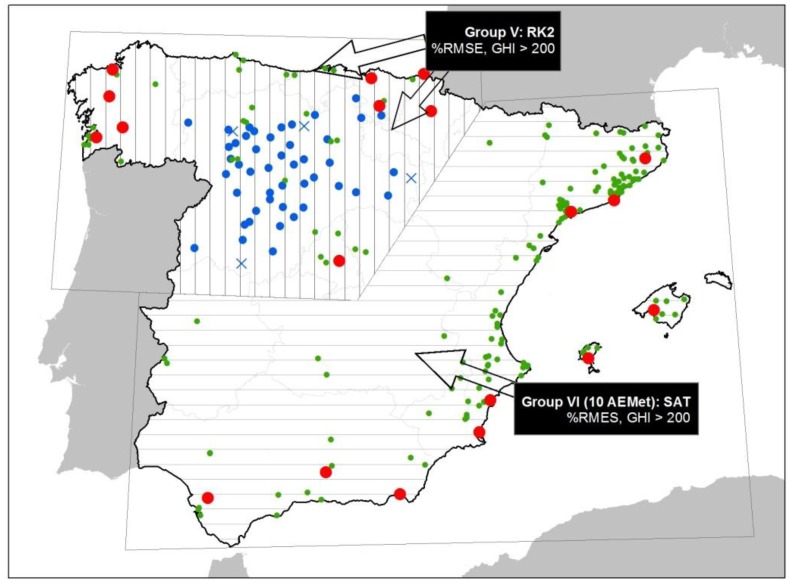
The best method study area and combination of two sub-groups.

Figure 8 presents the combination of the best method/group, with RK2 in Group V represented with a vertical line and SAT in Group VI by horizontal lines. The figure shows which stations are part of the interpolations for Group V, which are the AEMet stations (red symbols) plus the JCY stations (blue symbols). For both groups, the figure also shows the volunteer stations to be validated with their corresponding winning methods (green symbols).

After identifying the method/group to be applied to the entire study area (see Equation (10)), the volunteer stations were validated in view of two restrictions under which the methods of estimation present more accurate results. These restrictions are:

- Considering SR values with a GHI threshold >200: This was done based on the results of previous experiments (Section 4.2), in which it was found that for this threshold the methods of estimation present the best levels of error and standard deviations.
- Validation only on clear days according to the classification of the day type proposed by [61]. This classification uses the magnitude of the daily clearness index K_T_ [62] in Equation (10) and defines a clear day as one where K_T_ > 0.7 [63], with K_T_ defined as the ratio between the daily global horizontal radiation (H_G_^d^) and the daily extraterrestrial radiation on a horizontal surface (H_0_^d^):
  (10) $$K_{T}=\frac{H_{G}^{d}}{H_{0}^{d}}$$

H_G_^d^ from 15-minute GHI intervals was aggregated using trapezoidal integration on the R *pracma* package [64]. The underlying calculations with respect to H_0_^d^ procedures were carried out using the R *solaR* package [65].

**Figure 9.**
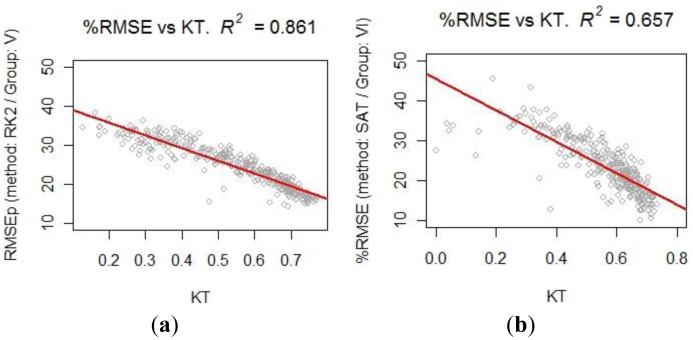
Relationship between K_T_ and daily %RMSE: (**a**) RK2: Group V (CYL + 9 AEMet); (**b**) SAT: Group VI (10 AEMet).

A strong negative correlation between K_T_ and %RMSE for the various methods of spatial GHI estimation has been found. The RK2 method in Group V presents a strong negative correlation of R = −0.928. The SAT method in Group VI also presents a strong correlation, with a value of R = −0.81. Figure 9 presents a scatter plot of K_T_ *vs* %RMSE, with its regression indexes shown here.

Figure 9a presents the case of the RK2 method in Group V, which shows a strong negative correlation of R = −0.928. Figure 9b presents the case of the SAT method in Group VI, which shows a negative average correlation of R = −0.878.

After applying the aforementioned conditions of validation, we proceeded to analyze the number of days in which the VWO stations presented error levels between the average, ±2 standard deviation (mean, 2 std-dev) and ±3 standard deviations (mean, 3 std-dev) with respect to the estimation models used.

Of the 31 VWO stations included in the RK2 method in Group V we found that:

- Eight stations (26%) presented a level within the 2 std-dev.
- Eleven stations (35%) presented a level within the 3 std-dev.

Of the 175 VWO stations included in the SAT method in Group VI we found that:

- 130 stations (74%) presented a level within the 2 std-dev.
- 135 stations (77%) presented a level within the 3 std-dev.

## 5. Conclusions

The selection of the best spatial estimation method for GHI (five in total) at 15-minute intervals, evaluated in this study depend on the density of stations that observe this variable over a determined area. When the area of interest is adequately covered by ground station sensors, the best method is Regression Kriging (RK), supported by GHI values derived from satellite images and the latitude of the stations' locations (RK2). The study found that by using this RK2 method it is possible to interpolate values of the GHI level-based beyond the 25 km limit stated by the reference bibliography. Even more, the RK2 method was tested until 108 km in which the %RMSE never reaches the 25% stated by the reference bibliography. Therefore, the RK2 method already developed throughout this study, draws on the strength of both sources of information which allows covering interpolation distances beyond the limit of only using the observations by surface stations but with an error level below of only values derived from satellite images.

These previous facts enable some practical applications. For example: (1) the validation of sensor GHI from VWO stations that was used in this study; (2) the future generation of GHI surface at 15-minute intervals in order to estimate the potential production of energy by the sun in places where there is no direct GHI observations and with an error level below the 25%RMSE.

However, the disadvantage of using RK2 is the need of GHI values derived from satellite images.

On the other hand, when the station sensors are very scattered, or when the work area is not very dense, then the best method (compared to other methods of interpolation) is to estimate GHI based on satellite images, a result consistent with those presented in the literature.

For any of the methods (Interpolations or Satellite Estimates), more outliers are found when trying to estimate GHI at times when GHI values are low (dawn/dusk). As greater GHI values are estimated, the std-dev and mean error decrease (the slope of variation over time decreases and the authors believe that the stations' clock drift problems are minimized).

In addition to the main objective, temporal interpolations had been carried out based on the average observations in a single station in order to: (i) synchronize them at minutes 0, 15, 30 and 45 per hour (independent from the source); (ii) eliminate data whenever there were more observations than necessary (usually at night). This temporal interpolation was carried out whenever the window of time between two contiguous observations was less than 30 min, these characteristics in our data avoid us to use other sophisticated and complex methods of interpolation.

The practical applications of the results of the present study, under clear sky conditions and for GHI values >200, made possible to determine that 75% (from a total of 206 stations) of the observations by SR sensors at the volunteer stations analyzed were within the margin of error for the models evaluated. These values should not be underestimated even though they were the result of a simple approach, since, in absolute values, this 67% represents 138 stations, which is twice of the 69 official stations used in the present study.

## 6. Future Works

The likelihood that 67% of the VWO stations will present valid values, should not be underestimated, since these stations could help improve spatial densification of GHI observations with surface sensors. This prompts us to initiate further researched focused directly on validating GHI observations for VWO stations in view of other factors that might influence in the interpolation of GHI such as the location of VWO stations (“Z” altitude of the station, surrounding relief and/or buildings, among other factors).

Another line of work to be explored is checking whether applying more advanced methods for generating time sequences such as the mean preserving algorithm proposed by [66], would somehow improve the generation of 15-minute time sequences for the CyL network, based on 30-minute observations, and how this might help make up for gaps in observations in the volunteer stations due to a lack of storage or interruptions in communications services.

## Figures and Tables

**Table 1.** Ranking of methods without discriminating irradiance values (daily aggregation) according to the six ways of grouping data: (AEMet + CYL); II (AEMet + 4 CYL); III (AEMet); IV (CYL); V (CYL + 9 AEMet); VI (10 AEMet).

**Score**	**I**		**II**		**III**		**IV**		**V**		**VI**		**Statistic**

***1****^st^*	**RK2**	**23.75**	**RK2**	**27.09**	**SAT**	**26.93**	**RK2**	**21.77**	**RK2**	**23.27**	**SAT**	**23.12**	%RMSE
***2****^nd^*	SAT	29.09	SAT	27.49	RK2	28.02	RK1	25.25	RK1	28.59	RK2	29.82	
***3****^rd^*	RK1	30.59	RK1	39.28	IDW	39.86	OK	25.41	IDW	28.86	IDW	34.16	
***4****^th^*	IDW	30.79	IDW	39.50	RK1	41.17	IDW	25.86	OK	28.92	OK	36.75	
***5****^th^*	OK	31.61	OK	40.56	OK	43.22	SAT	28.95	SAT	29.94	RK1	37.91	

**Table 2.** Ranking of methods for irradiance values greater than 200 (daily aggregation) according to the six ways of grouping data I (AEMet + CYL); II (AEMet + 4 CYL); III (AEMet); IV (CYL); V (CYL + 9 AEMet); VI (10 AEMet).

**Score**	**I**		**II**		**III**		**IV**		**V**		**VI**		**Statistic**

***1****^st^*	**RK2**	**17.45**	**SAT**	**18.78**	**SAT**	**18.29**	**RK2**	**16.50**	**RK2**	**17.37**	**SAT**	**17.06**	%RMSE
***2****^nd^*	SAT	19.90	RK2	19.18	RK2	19.97	RK1	19.04	SAT	20.63	RK2	21.98	
***3****^rd^*	RK1	22.25	RK1	27.50	IDW	28.38	OK	19.12	IDW	20.94	IDW	25.68	
***4****^th^*	IDW	22.49	OK	27.84	RK1	29.07	IDW	19.57	RK1	21.04	OK	26.32	
***5****^th^*	OK	22.73	IDW	27.96	OK	29.77	SAT	20.82	OK	21.18	RK1	28.56	

**Table 3.** Average distance in kilometers from nearest neighboring station and between all the stations for each group, standard deviation of average distance, number of stations, group area and station density.

**Group**	**Avg (NN) Distance (km)**	**Avg (Mean) Distance (km)**	**Avg (Std-dev)**	**Number of Stations**	**Group Area (km^2^)**	**Mean Area per Stations (km^2^)**

I (AEMet + CYL)	43.69	288.93	255.41	69	497,167	7,205
II (AEMet + 4 CYL)	100.16	470.30	288.45	23	497,167	21,615
III (AEMet)	97.43	495.86	298.45	19	497,167	26,166
IV (CYL)	25.94	137.42	97.24	50	94,226	11,884
V (CYL + 9 AEMet)	30.03	178.82	136.45	59	195,064	3,306
VI (10 AEMet)	111.26	373.37	268.3	10	303,839	30,383

**Table 4.** The best method per group and statistic, without differentiating between irradiance values (aggregate for the total number of days).

**Group**	**Best Method**	**Mean**	**Std-dev**	**% Days Excluded**	**% Days as N°1**	**Statistic**
I (CYL + AEMet)	RK2	23.75	11.38	3.35	91.62	%RMSE
II (AEMet + 4 CYL)	RK2	29.09	10.02	2.67	49.85	
III (AEMet)	SAT	26.93	10.03	3.86	69.44	
**IV (CYL)**	**RK2**	**21.77**	**11.57**	**0.00**	**84.27**	
V (CYL + 9 AEMet)	RK2	23.27	11.63	1.40	92.16	
VI (10 AEMet)	SAT	23.12	10.53	4.42	83.91	

**Table 5.** The best method by group and statistic, discriminating irradiance values greater than 200 (aggregate for the total number of days).

**Group**	**Best Method**	**Mean**	**Std-dev**	**% Days Excluded**	**% Days as N°1**	**Statistic**
I (CYL + AEMet)	RK2	17.45	7.23	3.07	81.01	%RMSE
II (AEMet + 4 CYL)	SAT	18.78	5.74	0.29	7 2.40	
III (AEMet)	SAT	18.29	5.35	1.78	84.27	
**IV (CYL)**	**RK2**	**16.50**	**7.96**	**0.28**	**74.16**	
V (CYL + 9 AEMet)	RK2	17.37	7.90	1.40	81.23	
VI (10 AEMet)	SAT	17.06	6.24	1.26	89.27	

**Table 6.** Statistics for the RK2 Method in Group IV (CYL).

**Threshold**	**%RMSE (%)**	**Std-dev %RMSE (%)**	**RMSE (W/m^2^)**	**Std-dev RMSE (W/m^2^)**	**RMSE Range (W/m^2^)**	**%RMSE (All)**	**Std-dev %RMSE (All)**
NA	22.46	2.87	68.95	10.36	59–79	247.31	508.06
0.1	22.46	2.87	68.95	10.36	59–79	148.74	362.46
10	21.03	2.75	68.97	10.59	59–81	51.90	25.22
50	17.51	2.45	72.86	11.51	61–84	32.73	9.15
**200**	**11.26**	**1.35**	**75.21**	**12.97**	**62–88**	**17.88**	**3.23**

**Table 7.** Statistics for the RK2 method in Group V (CYL + 9 AEMet).

**Threshold**	**%RMSE**	**Std-dev %RMSE**	**RMSE (W/m^2^)**	**Std-dev RMSE (W/m^2^)**	**RMSE Range (W/m^2^)**	**%RMSE (All)**	**Std-dev %RMSE (All)**
NA	24.70	4.26	71.23	23.38	48–95	533.20	1,114.30
0.1	24.69	4.26	71.23	23.38	48–95	357.53	933.02
10	22.90	3.83	70.49	22.96	48–93	63.97	53.70
50	18.95	3.22	68.95	19.11	50–88	37.98	17.61
200	12.29	2.01	63.39	12.25	51–76	22.42	13.76

**Table 8.** Statistics for GHI estimates derived from satellite images in Group VI (10 AEMet).

**Threshold**	**%RMSE**	**Std-dev %RMSE**	**RMSE (W/m^2^)**	**Std-dev RMSE (W/m^2^)**	**RMSE Range (W/m^2^)**	**%RMSE (All)**	**Std-dev %RMSE (All)**
NA	22.85	0.87	105.13	15.67	89–121	713.09	823.02
0.1	22.84	0.87	105.13	15.68	89–121	500.27	476.25
10	21.55	1.03	104.64	15.78	89–120	84.05	33.26
50	18.04	1.27	103.59	15.12	88–119	46.06	8.14
200	11.46	1.25	101.21	15.91	85–117	23.13	2.64
